# Evaluation of Browning Markers in Subcutaneous Adipose Tissue of Newly Diagnosed Gastrointestinal Cancer Patients with and without Cachexia

**DOI:** 10.3390/cancers14081948

**Published:** 2022-04-12

**Authors:** Alessio Molfino, Roberta Belli, Giovanni Imbimbo, Raffaella Carletti, Maria Ida Amabile, Federica Tambaro, Cira R. T. di Gioia, Elena Belloni, Elisabetta Ferraro, Giuseppe Nigri, Maurizio Muscaritoli

**Affiliations:** 1 Department of Translational and Precision Medicine, Sapienza University of Rome, 00185 Rome, Italy; roberta.belli@uniroma1.it (R.B.); giovanni.imbimbo@uniroma1.it (G.I.); raffaella.carletti@uniroma1.it (R.C.); federica.tambaro@uniroma1.it (F.T.); maurizio.muscaritoli@uniroma1.it (M.M.); 2 Department of Surgical Sciences, Sapienza University of Rome, 00161 Rome, Italy; mariaida.amabile@uniroma1.it; 3 Department of Radiological, Oncological and Pathological Sciences, Sapienza University of Rome, 00161 Rome, Italy; cira.digioia@uniroma1.it; 4 Department of Medical-Surgical Sciences and Translational Medicine, Sapienza University of Rome, 00189 Rome, Italy; elena.belloni@uniroma1.it (E.B.); giuseppe.nigri@uniroma1.it (G.N.); 5 Department of Biology, University of Pisa, 56127 Pisa, Italy; elisabetta.ferraro@unipi.it

**Keywords:** cachexia, cancer, browning, subcutaneous adipose tissue, wasting

## Abstract

**Simple Summary:**

Cachexia occurs frequently in cancer patients with deep metabolic derangements. The browning of adipose tissue promotes thermogenesis and energy expenditure and, in cancer, has been considered a major determinant of adipose tissue atrophy. We evaluated the molecular phenotype of this phenomenon in the subcutaneous adipose tissue (SAT) of newly diagnosed gastrointestinal cancer patients compared to controls. We observed that the modulation of different markers of the browning of SAT in gastrointestinal cancer and, in particular, pancreatic cancer showed significant changes in UCP1 and PGC1α; PGC1α was highly expressed in cachectic patients. Our study highlights the relevance of browning in patients with cancer, in particular in those with pancreatic cancer. Understanding the browning phenomenon may allow us to counteract these metabolic alterations before the development of severe cachexia, which is characterized by deep adipose and muscle depletion, negatively affecting survival and quality of life.

**Abstract:**

We assessed the molecular phenotype of the browning of white adipose tissue in newly diagnosed cancer patients and controls undergoing surgery for gastrointestinal tumors and for non-malignant diseases, respectively. We collected subcutaneous adipose tissue (SAT) samples and using RT-PCR, we analyzed the expression of markers of browning and using Western blot the protein levels of UCP1 and PGC1α. The *Ucp1* mRNA levels were lower in cancer patients vs. controls (*p* = 0.01), whereas *Cidea* and *Tmem26* mRNA levels were higher in cancer patients. We found higher PGC1α protein levels in patients vs. controls, while no differences were seen for UCP1. The *Ucp1* expression was lower in cachectic and non-cachectic patients vs. controls, whereas *Cidea* expression was higher in cachectic and non-cachectic patients vs. controls. *Pgc1α* mRNA levels were higher in cachectic vs. non-cachectic patients (*p* = 0.03) vs. controls (*p* = 0.016). According to type of tumors, we did not observe differences in *Cidea* expression, whereas *Pgc1α* was higher in pancreatic cancer vs. colorectal and vs. controls. We observed the lower expression of *Ucp1* in pancreatic and colorectal cancer vs. controls. We documented higher UCP1 protein levels in pancreatic cancer patients vs. colorectal (*p* = 0.002) and vs. controls (*p* = 0.031). PGC1α protein levels were higher in pancreatic cancer patients vs. controls. Different markers of the browning of SAT are modulated, and pancreatic cancer showed changes in UCP1 and PGC1α; PGC1α was highly expressed in cachectic patients, with clinical implications that should be further clarified.

## 1. Introduction

Cachexia occurs frequently in cancer patients and is associated with metabolic alterations involving central mechanisms and peripheral tissues, leading to body weight loss [1,2,3]. This implies deep changes, including wasting, mainly of adipose tissue and muscle mass [1,2,4] representing predictors of poor clinical outcomes [4,5,6].

Adipose tissue is considered pivotal in the development of cachexia in cancer patients, and different mechanisms of adipose atrophy were identified, including increased lipolysis and the enhanced browning of white adipose tissue [7,8,9]. 

Browning is a metabolic process characterized by the emergence of beige adipocytes in white adipose tissue (WAT), promoting thermogenesis and energy expenditure [10]. In cancer, browning has been considered a major determinant of adipose tissue atrophy, and the inhibition of specific pathways, such as that of parathyroid-hormone-related protein (PTHrP), improved cachexia in an experimental model [8]. Importantly, several biomarkers of browning were identified in previous animal studies that allowed a phenotypic characterization of the presence of beige/brown adipocytes in these settings [11,12]. In particular, animal and human experiments documented the modulation of specific molecules including *Tbx1, Eva, Dio2, Tmem26* and *Cidea*, representing the signature of beige-selective and brown-selective genes [11,13].

Petruzzelli et al. showed that the browning process may anticipate skeletal muscle wasting in cancer and is associated with enhanced uncoupling protein 1 (UCP1) expression [13].

We recently showed that gastrointestinal cancer patients with cachexia showed decreased adipocyte size, increased fibrosis and inflammatory changes in subcutaneous adipose tissue and that histological changes reflected alterations in adiposity evaluated using CT scan [14].

In the present study, we aimed to (i) assess the gene expression and the protein levels of the markers of browning in the subcutaneous adipose tissue (SAT) of patients with gastrointestinal cancer with and without cachexia before any anticancer treatment, including surgery, compared to non-cancer, non-cachectic patients and to (ii) evaluate potential differences in these markers between different types of gastrointestinal cancer.

## 2. Materials and Methods

### 2.1. Participant’s Selection

This was an observational, controlled study performed on gastrointestinal cancer patients eligible for surgery and on controls enrolled at the Department of Medical-Surgical Sciences and Translational Medicine, Sapienza University of Rome, Italy. The study was performed in accordance with the Declaration of Helsinki and approved by the local Ethics Committee (Sapienza University, Azienda Sant ’Andrea Hospital, Rome, Italy—prot. n. 167SA_2017). All patients provided written informed consent to participate in the study.

We enrolled patients with a new diagnosis of pancreatic, gastric or colorectal cancer eligible for surgical tumor resection, naïve to any anticancer treatments, including adjuvant therapy, and subjects with non-malignant diseases undergoing abdominal surgery, serving as controls. 

We included patients with age ≥ 18 years and with the ability to give informed consent. In both groups, we excluded patients with acute or chronic conditions negatively affecting nutritional status, including infections, heart failure, liver cirrhosis, chronic kidney diseases and clear signs of malabsorption or intestinal occlusion. We also excluded patients with dysphagia.

### 2.2. Clinical, Nutritional Status Evaluation and Adipose Tissue Biopsy

In all the participants, we registered information on nutritional status including current weight, usual weight and involuntary body weight loss in the prior 6 months, and we calculated body mass index (BMI). In cancer patients, in accordance with international criteria routinely used in clinical studies [1,4], cachexia was diagnosed as a non-volitional body weight loss >5% or BMI <20 kg/m^2^ and any degree of weight loss >2% [15], assessed during a study visit before surgery. During the enrollment in a fasting state, we collected blood samples in EDTA tubes, and after centrifugation, we measured albumin and C-reactive protein (CRP) circulating levels, and we tested hemoglobin concentration with standard automated techniques. Moreover, from patient clinical records, we collected data on the staging and histology of the cancer and the number and type of comorbidities.

During the first phase of the surgical procedure, in both cancer patients and controls, we collected the specimens of SAT (approximately 1 cm^3^) obtained anteriorly to the anterior sheath of the rectus abdominis. The samples were immediately frozen in liquid nitrogen and in part included in OCT, and stored at −80 °C.

### 2.3. Quantitative Real-Time PCR

We examined the expression of brown/beige selective genes of the adipose tissue, as well as those involved in the metabolic change of white adipocytes into beige cells (the browning of WAT) (Table 1), as previously described by others [11,12,13], to reveal potential differences between gastrointestinal cancer patients and controls. The primers we used, according to the available literature [16,17], are shown in Table 1. 

Total RNA was extracted from subcutaneous white adipose tissue using an RNeasy Lipid Tissue Mini Kit (Qiagen, Germantown, MD, USA) according to the manufacturer’s instructions. cDNA was synthetized from 250 ng of total RNA using the High-Capacity cDNA Reverse Transcription Kits (Applied Biosystems, Thermo Fisher Scientific, Wilmington, DE, USA), according to the manufacturer’s instructions. Comparative real-time PCR was performed with GoTaq^®^ qPCR Master Mix (Promega, Madison, WI, USA), using Applied Biosystem 7900HT Fast. Data were normalized to β-Actin (calibrator), used as the internal control. Resulting data were analyzed using SDS2.4 Software (Applied Biosystems, Bedford, MA, USA), and fold-change was determined by using the 2^−ΔΔCT^ [18]. All reactions were performed in duplicate. The primers we used are shown in Table 1.

### 2.4. Protein Isolation and Western Blot

Subcutaneous adipose tissue (SAT) was lysed using ice-cold RIPA buffer (50 mM Tris/HCl pH 8.1% Triton X, 150 mM NaCl, 0.5% sodium-deoxycholate, 0.1% SDS) supplemented with Phosphatase Inhibitor Cocktail 2 and 3 (Sigma-Aldrich, Dorset, UK) and Halt Protease Inhibitor (Thermo Scientific, Waltham, MA, USA). A clear supernatant was obtained via the centrifugation of lysates at 13,000 rpm for 20 min at 4 °C. Protein concentrations were measured using the Bradford protein assay (Bio-Rad). Western blot analysis was performed by loading and separating aliquots of the total lysate via SDS-PAGE using Miniprotean precast gels (Bio-Rad, Portland, ME, USA), and proteins were transferred to nitrocellulose membranes (Bio-Rad) using a Trans-Blot semidry electrophoretic system (Bio-Rad). Membranes were blocked for 1 h at RT with 5% non-fat milk in T-TBS (Tris-Buffered Saline with 0.05% Tween 20). Incubation with primary specific antibodies was performed in blocking solution overnight at 4 °C, and incubation with horseradish peroxidase-conjugated secondary antibodies or alkaline phosphatase (AP)-conjugate secondary antibodies was performed in blocking solution for 1 h at RT. The following primary antibodies were used for detection: PGC1α (#3242, Millipore) 1:1000, UCP1 (#GTX112784, GeneTex) 1:1000, actin-β (#A2228, Sigma-Aldrich, Dorset, UK) 1:1000. Specific antibody signals were detected using appropriate horseradish peroxidase-conjugated secondary antibody anti-mouse (#1706516, Goat Anti-Mouse IgG antibody, Bio-Rad) 1:8000 or AP-conjugate secondary antibody anti-rabbit (#A16099, Invitrogen, Carlsbad, CA, USA) 1:3000. Immunoreactive bands were visualized using SuperSignal West Pico Plus Chemiluminescent Substrate (Thermo Scientific, Waltham, MA, USA) and using the NBT/BCIP Color Development Substrate system (#S3771, Promega, Madison, WI, USA) in AP buffer (0.1 M TrisHCl pH 9.5, 100 mM NaCl, 5 mM MgCl_2_). Then, 0.5 M EDTA (pH 8.0) was used to block the reaction. Protein expression was normalized for β-Actin protein levels. The relative amounts of each band were quantified with densitometry using the ImageJ software.

### 2.5. Immunohistochemical Evaluation of UCP1 in Adipose Tissue

UCP1 localization was evaluated with immunohistochemical stains performed on frozen adipose tissue sections (5 μm). Endogenous peroxidase activity was blocked using 3% hydrogen peroxide. The sections were incubated at room temperature for 1 h with anti-human antibody UCP1, (1:750, rabbit polyclonal antibody, GTX112784 Gene Tex, Irvine, CA, USA). The Universal Quick Kit, Peroxidase, R.T.U. Staining System (Vector laboratories, Burlingame, CA, USA) was used to label the primary antibody. The reaction product was visualized with 3,3′-diaminobenzidine (DAB) (Vector laboratories, Burlingame, CA, USA) and counterstaining with Mayer hematoxylin. A negative control was obtained by omitting the primary antibody. The immunostained slides of adipose tissue sections were viewed by two independent pathologists using a Leica microscope (Leitz Camera) [19].

### 2.6. Statistical Analyses

Patients’ characteristics were described using mean ± SD and median (25th; 75th percentiles) for non-normally distributed variables. Normal distribution was tested using the Shapiro–Wilk test. Categorical variables were described as number (%). To analyze potential differences between cancer patients with cachexia, cancer patients without cachexia and controls, we performed the Analysis of Variance (ANOVA) and the Kruskal–Wallis test, as appropriate. According to the normal/non-normal distribution, we also assessed differences between groups using the two-tailed t-test or the Mann–Whitney test, respectively. The chi-square test was used to verify association(s) between categorical variables. A *p* value < 0.05 was considered statistically significant. SPSS version 26 was used to perform statistical analyses. 

## 3. Results

### 3.1. Patient’s Characteristics 

We enrolled 24 gastrointestinal cancer patients and 13 non-oncology patients who underwent surgery for non-malignant diseases (i.e., cholecystectomy for gallstones, abdominal wall surgery for hernia and surgery for cysts). The clinical characteristics of the participants are summarized in Table 2. In particular, gastrointestinal cancer patients (13 females and 11 males)—9 pancreatic, 7 gastric and 8 colorectal—presented with a median age of 74 years (IQR 68; 80) and a mean BMI of 27.04 ± 3.29 kg/m^2^, whereas the control group (7 females and 6 males) presented with a median age of 63 years (IQR 55; 66) and a mean BMI of 28.18 ± 4.45 kg/m^2^ (Table 2).

### 3.2. Nutritional and Inflammatory Status

In gastrointestinal cancer patients, we documented a mean body weight loss of 4.85 ± 3.30%, and cachexia accounted for 38% (9/24) (Table 2).

No modifications in body weight in the prior six months were recorded in the control group (Table 2). We did not observe differences in body weight loss (%) and BMI among the different types of cancer patients (*p* = 0.460 and *p* = 0.518, respectively). Although CRP serum levels were not different among patients and controls, only the cancer group showed median CRP values above the normal range (0–0.5 mg/dL) (Table 2). 

### 3.3. Browning Genes Expression and Evaluation of UCP1 and PGC1a Protein Levels in SAT of Gastrointestinal Cancer Patients and Controls

**Figure 1:**
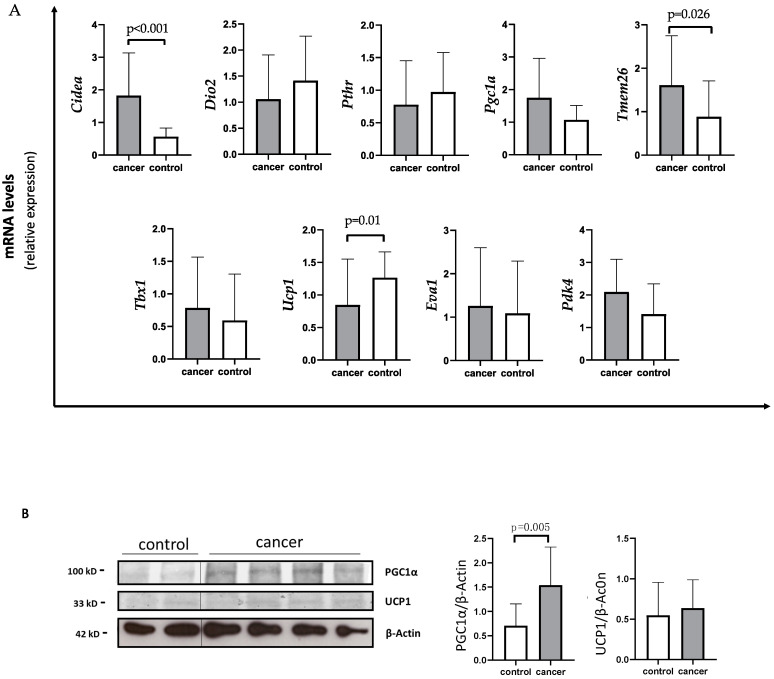
Evaluation of browning genes and protein expression in white adipose tissue (WAT) of gastro-intestinal cancer patients. (**A**) The mRNA levels of Cell Death-Inducing DFFA-Like Effector A (*Cidea*), Iodothyronine Deiodinase 2 (*Dio2*), parathyroid hormone receptor (*Pthr*), peroxisome proliferator-activated receptor gamma coactivator-1-α (*Pgc1α*), Transmembrane protein 26 (*Tmem26*), T-box transcription factor 1 (*Tbx1*), uncoupling protein 1 (*Ucp1*), EVA1 and Pyruvate Dehydrogenase Kinase 4 (*Pdk4*) were analyzed using quantitative real-time PCR in SAT of gastrointestinal cancer patients (*N* = 24) and controls (*N* = 13). (**B**) Representative Western blot images and protein densitometry quantification for PGC1α and UCP1 protein levels in SAT of a subset of cancer patients (*N* = 19) and controls (*N* = 8). β-Actin was used as loading control.

Using quantitative PCR (qPCR) analysis, gastrointestinal cancer patients showed a significant decreased expression of *Ucp1* compared to controls (median 0.56 IQR 0.32; 1.19 vs. 1.17 IQR 0.85; 1.67) (*p* = 0.01) (Figure 1A).

Additionally, cancer patients showed higher *Cidea* levels with respect to controls (median 1.34 IQR 0.96; 2.41 vs. 0.60 IQR 0.44; 0.86) (*p* < 0.001) (Figure 1A). In parallel, *Tmem26* levels were significantly increased in cancer patients compared to controls (median 1.51 IQR 0.76; 2.03 vs. 0.77 IQR 0.41; 0.99) (*p* = 0.026) (Figure 1A). Additionally, *Pdk4* levels tended to increase in the SAT of cancer patients with respect to controls (mean 2.09 ± 1.00 vs. 1.46 ± 0.90) (*p* = 0.066) (Figure 1A), whereas no changes in *Pgc1α* expression were detected in cancer patients with respect to controls (median 1.40 IQR 0.80; 2.45 vs. 1.17 IQR 0.68; 1.42) (*p* = 0.179). However, using Western blot analysis performed in a subset of samples, we found higher PGC1α levels in gastrointestinal cancer patients with respect to controls (1.50 IQR 0.97; 1.71 vs. 0.73 IQR 0.42; 1.12) (*p* = 0.005) (Figure 1B), whereas no difference was observed between gastrointestinal cancer patients and controls in UCP1 protein levels (Figure 1B).

### 3.4. Evaluation of Browning Gene Expression, UCP1 and PGC1a Protein Levels in SAT of Gastrointestinal Cancer Patients with and without Cachexia and in Controls

**Figure 2:**
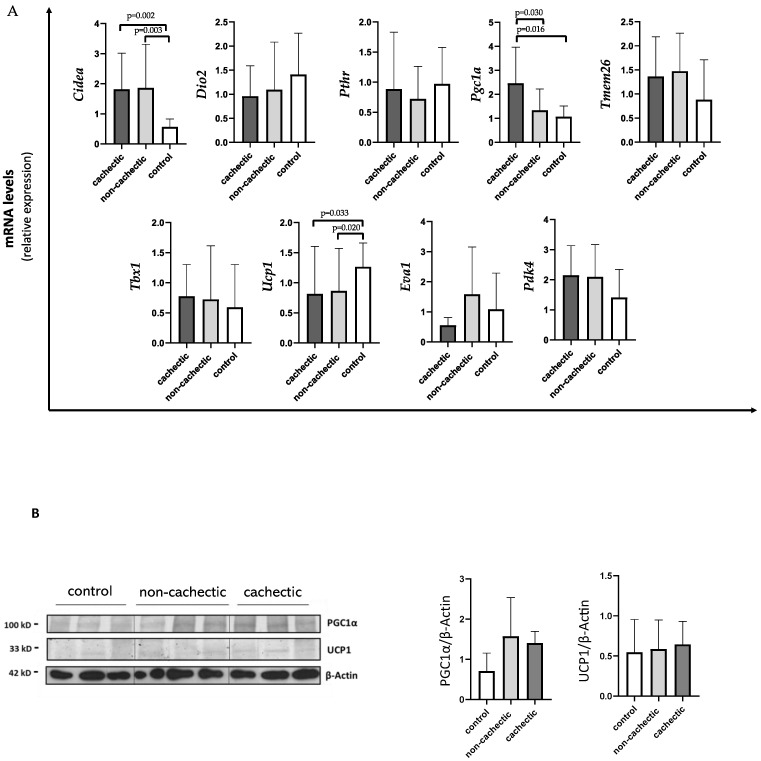
Evaluation of browning genes and protein expression in SAT of cachectic and non-cachectic cancer patients.(**A**) The mRNA levels of C*idea, Dio2, Pthr, Pgc1α, Tmem26, Tbx1, Ucp1, Eva1* and *Pdk4* were analyzed using quantitative real-time PCR in cachectic (*N* = 9) and non-cachectic (*N* = 15) gastrointestinal cancer patients and in control group (*N* = 13) (**B**) Representative Western blot images and protein densitometry quantification for PGC1α and UCP1 protein levels in SAT of a subset in cachectic (*N* = 7) and non-cachectic (*N* = 12) patients and in control group (*N* = 8). β-Actin was used as loading control.

As shown in Figure 2A, *Ucp1* expression levels were significantly decreased in cachectic (median 0.56 IQR 0.35; 1.39) and non-cachectic (median 0.61 IQR 0.26; 0.71) cancer patients compared to controls (median 1.17 IQR 0.85; 1.67) (*p* = 0.033 and *p* = 0.020, respectively). We also found a significant increase in *Cidea* expression in cachectic patients vs. controls (1.40 IQR 0.94; 2.49 vs. 0.60 IQR 0.44; 0.86) (*p* = 0.002), as well as in non-cachectic patients (median 1.17 IQR 0.96; 2.47) vs. controls (*p* = 0.003) (Figure 2A). For both *Ucp1* and *Cidea* expression, we did not find differences between cachectic and non-cachectic patients (Figure 2A).

Interestingly, cachectic patients showed higher expressions of *Pgc1α* (median 2.25 IQR 1.22; 3.47) compared to non-cachectic patients (median 1.04 IQR 0.69; 1.94) and to controls (median 1.17 IQR 0.68;1.42) (*p* = 0.030 and *p* = 0.016, respectively) (Figure 2A).

Moreover, no differences between cachectic and non-cachectic patients were seen in *Tmem26* mRNA expression, nor in *Pdk4* (Figure 2A). 

In Western blotting analysis performed in a subset of samples, we did not find differences in PGC1α and UCP1 protein levels among the groups (cachectic and non-cachectic patients and controls) (Figure 2B).

### 3.5. Differences in Browning Gene Expression and Protein Levels according to the Type of Gastrointestinal Cancer

**Figure 3:**
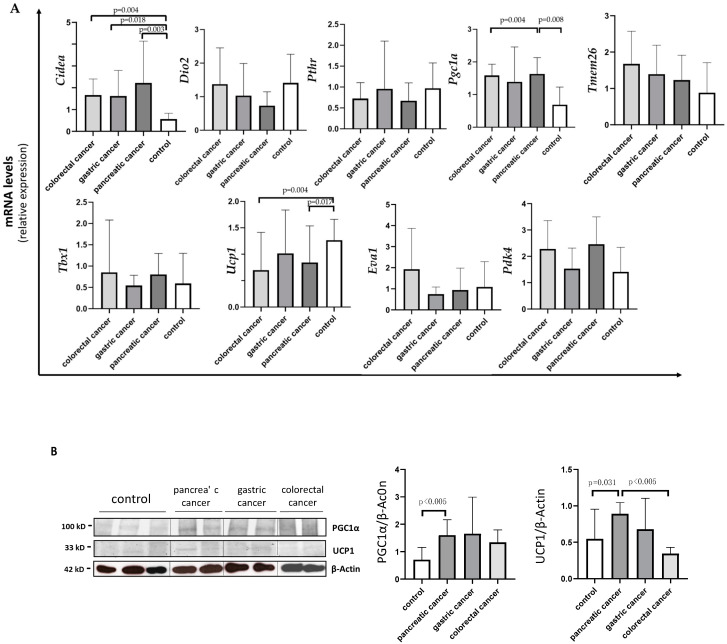
Evaluation of browning genes and protein expression in white adipose tissue (WAT) of cancer patients divided by the three types of gastrointestinal cancer (colorectal, stomach and pancreas). (**A**) The mRNA levels of *Cidea, Dio2, Pthr, Pgc1α, Tmem26, Tbx1, Ucp1, Eva1* and *Pdk4* were analyzed using quantitative real-time PCR in SAT of gastrointestinal cancer patients according to the different type of tumor (gastric *N* = 7, colorectal *N* = 8, pancreas *N* = 9) and in control group (*N* = 13). (**B**) Representative Western blot images and protein densitometry quantification for PGC1α and UCP1 in a subset of pancreatic (*N* = 7), gastric (*N* = 5) and colorectal (*N* = 7) cancer patients, and in control group (*N* = 8). β-Actin was used as loading control.

Analyzing *Cidea*, we observed increased expression in all the three types of cancer compared to controls: colorectal (median 1.71 IQR 1.00; 2.18, *p* = 0.004); gastric (median 1.12 IQR 0.91; 2.47, *p* = 0.018); pancreatic (median 1.40 IQR 0.86; 3.10, *p* = 0.003) (Figure 3A). No differences were detected in *Cidea* mRNA levels among the three different types of gastrointestinal cancer.

Analyzing *Pgc1α*, we found higher expression in pancreatic cancer patients (median 2.08 IQR 1.43; 3.47) vs. controls (median 1.17 IQR 0.68; 1.42) (*p* = 0.004) and vs. colorectal cancer patients (median 0.73 IQR 0.49; 1.47) (*p* = 0.008), and we found a similar trend, although not significant, vs. gastric patients (*p* = 0.159) (Figure 3A). 

Analyzing *Ucp1*, we found lower expression in pancreatic (median 0.63 IQR 0.35; 1.57) and colorectal (median 0.35 IQR 0.31; 1.07) cancer patients compared to controls (median 1.26 IQR 0.92; 1.81) (*p* = 0.017 and *p* = 0.004, respectively) (Figure 3A).

**Figure 4:**
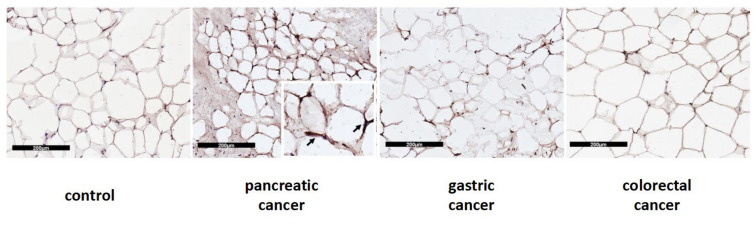
Immunohistochemical cytoplasmatic localization of UCP1 protein in SAT from control and patients with pancreatic, gastric and colorectal cancer. The scale bar used is 200 µm.

In Western blot analysis performed in a subset of samples, we documented significantly higher UCP1 protein levels in pancreatic cancer patients when compared to colorectal cancer patients (median 1.00 IQR 0.73; 1.11 vs. 0.36 IQR 0.31;0.41) (*p* = 0.002) and to controls (median 0.37 IQR 0.30; 1.00) (*p* = 0.031) (Figure 3B). Additionally, we described UCP1 localization in adipocytes among the different types of gastrointestinal cancers and controls, which is represented in Figure 4.

Finally, PGC1α protein levels were significantly higher in pancreatic cancer patients vs. controls (median 1.58 IQR 1.15; 2.06 vs. 0.73 IQR 0.42; 1.12, *p* = 0.004) (Figure 3B), whereas no differences were observed among the other cancer groups.

UCP1 protein is presented as a brownish crescent around unilocular adipocytes.

## 4. Discussion

In the present study, we were able to document the involvement of several changes in browning markers among patients with gastrointestinal cancer undergoing surgery. In particular, unexpectedly, *Ucp1* mRNA levels were shown to be decreased in our cohort of cancer patients with respect to controls, as well as among cachectic and non-cachectic patients compared to controls. 

UCP1 is located in the inner membrane of mitochondria and is considered central in the browning process due to its role in thermogenesis through the dissipation of proton gradients, producing heat instead of ATP [20]. In particular, PTHrP induced UCP1 expression, promoting cachectic phenotypes in a Lewis lung carcinoma (LLC) model of cancer cachexia [8]. However, recent reports have shown contradictory results regarding UCP1 expression in adipose tissue in cancer. In fact, although *Ucp1* gene expression and protein level were found to be increased in cancer in different animal and human studies [12,13], Michaelis K. et al. showed decreased UCP1 levels in a murine model of pancreatic-cancer-associated cachexia [21]. 

In particular, cachexia and low *Ucp1* expression in brown and white adipose tissue were associated with increased inflammation in the central nervous system and in the periphery and with anemia and changes in circulating testosterone levels [21]. 

Importantly, Rohm et al. [22] showed that in cancer cachexia mice models, the thermogenesis mediated by UCP1 may be less pivotal in driving the wasting process with respect to the results obtained in previous studies [8,13,23]. In particular, data showed that an AMP-activated protein kinase (Ampk)-stabilizing peptide determined the amelioration of adipose tissue wasting interfering with CIDEA pathways independently of UCP1; moreover, the absence of UCP1 did not protect against the development of cachexia in mice [23]. 

Interestingly, different thermogenic pathways independent of UCP1 were described to play a potential role in cancer cachexia [24]. However, data on independent thermogenic pathways of UCP1 are lacking in this setting, especially in humans. 

Our data suggest negative modulation in the *Ucp1* gene expression, and although not confirmed at the protein level, the data represent results that are, at least in part, in line with previous reports [21,22]. On the other hand, UCP1 protein levels were increased in pancreatic cancer patients, suggesting potential translational regulation according to the different cancer type.

In our analyses, *Cidea* and *Tmem26* mRNA levels and PGC1α protein levels were increased in the SAT of cancer patients with respect to controls, whereas *Pdk4* expression was only slightly increased with respect to controls. 

In particular, *Cidea* expression was higher among cachectic and non-cachectic patients vs. controls, whereas no difference was found between the cachectic and non-cachectic groups. CIDEA was recently indicated as a crucial factor for the development of adipose tissue wasting via the promotion of browning and increased lipolysis [23,25]. In particular, robust data showed that CIDEA regulates UCP1 for britening and thermogenesis in human adipocytes [25].

CIDEA expression was particularly increased in the adipocytes of cachectic cancer patients likely due to their reduced body size and low adiposity, determined by the presence of a poor nutrition status [26]. This phenomenon might not have been detectable in our cohort among cachectic and non-cachectic patients considering the relatively early stage of cancer disease (new diagnosis) and the limited percent of body weight loss experienced in the prior months. However, *Cidea* represents an important marker of browning contributing to adipose loss with mechanism(s) that may differ according to the type of cancer and/or patients’ characteristics (e.g., percent of body weight loss), as well as *Tmem26*, which was modulated in cancer but not according to the presence/absence of cachexia. 

Interestingly, regarding the differences between cachectic and non-cachectic cancer patients, we found that *Pgc1α* gene expression was higher in the first group, whereas no difference was found at the protein level. PGC1α is a key regulator of mitochondrial biogenesis. Interestingly, the irisin–PGC1α pathway was recently considered as a promoter of the browning process in adipose tissue [27]. In fact, PGC1α was initially suggested as a co-activator of PPAR- γ that regulates the expression of UCP1 and thermogenesis in brown adipocytes [28]. Lately, data showed that it stimulates the secretion of irisin from muscle able to interact with other tissues, including adipose [27,29]. PGC1α also represents a target of adaptative thermogenesis in brown adipose tissue by the activation of adrenergic receptor [30,31]. Studying PGC1α mechanisms may elucidate the metabolic regulation of adipose tissue in catabolic states, such as cancer.

We were not able to document significant changes in other makers of browning, including *Tbx1, Pthr, Eva1* and *Dio2*, in the SAT of our cohort patients, although previous experiments documented the modulation of these markers during the browning process [8,11,12,13]. In light of this, additional evidence is needed, especially in humans, to clarify their role.

Furthermore, our study allows us to speculate on the potential differences in browning markers according to the type of gastrointestinal cancer. In fact, we found that *Ucp1* mRNA levels were lower in colorectal and pancreatic cancer patients vs. controls, and the UCP1 protein level was higher in pancreatic cancer patients vs. controls and vs. colorectal cancer patients. This behavior was not observed for *Cidea* (no difference according to the type of tumor) but was similar for PGC1α, which showed significant modulation in pancreatic but not in other gastrointestinal cancers. 

Our observations appear to be clinically relevant considering that nutritional and metabolic alterations have a negative impact on prognosis in patients with gastrointestinal tumors, in particular in those with pancreatic cancer [32]. This is particularly important when taking into account that pancreatic cancer is a disease more frequently complicated by cachexia with respect to other gastrointestinal cancers [1,33]. 

The most common conditions complained about by patients are the high percentage of unvoluntary body weight loss and low appetite [4,34,35]. Recent evidence showed that approximately 70% of pancreatic cancer patients were affected by moderate or severe protein-energy malnutrition, conditioning a poor functional status [32]. In a model of cancer cachexia, low food intake was not the main reason determining weight loss; rather, changes in the expression of factors controlling lipid metabolism and thermogenesis in brown adipose tissue were involved in the adipose tissue catabolism in disease-associated cachexia [36]. 

Recent clinical observations indicate that patients with advanced pancreatic cancer lose a large proportion of visceral and subcutaneous adipose tissue rapidly (approximately in 4 weeks), and this was associated with poor survival [37]. 

We believe that the early identification of molecular targets involved in adipose tissue depletion, in particular the browning phenomenon, may allow us to identify the metabolic alterations observed in cachexia before developing a severe clinical picture represented by the deep adipose depletion observed in body composition analysis, which also negatively impinges on quality of life [1,12].

Our study presents several limitations, including the relatively reduced number of patients with each cancer type, the stage of cancer disease (i.e., first cancer diagnosis), which may have limited the presence of advanced cachexia likely associated with more evident adipose tissue depletion. However, this allowed us to avoid interference with other factors, in particular with anti-cancer treatments, on the catabolic state and to identify the role of the browning process in cachexia due to the early stage of cancer disease. Some of the browning markers showed non-univocal behavior in the gene expression and at the protein level, although this may be commonly observed in such studies. A second PCR primer or a different control would have been helpful to rule out potential experimental biases, although here, this was not performed. We did not assess browning markers in serum/plasma but specifically in SAT. Additionally, we did not perform the immunohistochemistry of PGC1α. To interpret the role of the browning markers in cachexia in a more complete manner, we believe that the study of the lipolytic pathways might have clarified the interaction with the browning process in the development of adipose tissue depletion. 

## 5. Conclusions

In conclusion, we found that in patients with gastrointestinal cancer, different markers of browning are modulated and, in particular, pancreatic cancer showed significant changes in terms of UCP1 and PGC1α. This suggests that thermogenesis abnormalities are clinically relevant in cancer cachexia and should be promptly investigated to counteract the development of severe nutritional and metabolic derangements occurring in cancer, which negatively impact patients’ prognoses.

## Figures and Tables

**Table 1:** Primers used for comparative real-time PCR.

*Cidea*	Fw 5′-GGAGCTCATCAGCAAGACTCTG-3′
	Rv 5′-AACTCTTCTGTGTCCACCACG-3′
*Ucp1*	Fw 5′-GCAGGGAAAGAAACAGCACCT-3′
	Rv 5′-ACTTTCACGACCTCTGTGGG-3′
*Pgc1* *α*	Fw 5′-CCTGCATGAGTGTGTGCTCT-3′
	Rv 5′-CAGCACACTCGATGTCACTCC-3′
*Tbx1*	Fw 5′-ACGACAACGGCCACATTATTC-3′
	Rv 5′-CCTCGGCATATTTCTCGCTATCT-3′
*Tmem26*	Fw 5′-ATGGAGGGACTGGTCTTCCTT-3′
	Rv 5′-CTTCACCTCGGTCACTCGC-3′
*Eva1*	Fw 5′-GGAATCCTGAGCGGTACGATG-3′
	Rv 5′-CTGGCAGGTGTATGTCCCATT-3′
*Dio2*	Fw 5′-ATGCTGACCTCAGAGGGACT-3′
	Rv 5′-ATCCTCACCCAATTTCACCTGT-3′
*Pthr*	Fw 5′-CGTTAGTTTCCGTCTCCACCTT-3′
	Rv 5′-GCAGAAATCCACACAGCTGA-3′
*Pdk4*	Fw 5′-GGAGCATTTCTCGCGCTACA-3′
	Rv 5′-ACAGGCAATTCTTGTCGCAAA-3′
*β-Actin*	Fw 5′-CCTGGCACCCAGCACAA-3′
	Rv 5′-GGGCCGGACTCGTCATA-3′

**Table 2:** Participants’ characteristics.

Clinical Parameter	Cancer Patients (*N* = 24)	Controls (*N* = 13)	*p*-Value
Age, y	74 (68; 80)	63 (55; 66)	0.0006
Male, n (%)	11 (46)	6 (46)	0.985
BMI, kg/m^2^	27.04 ± 3.29	28.18 ± 4.45	0.190
Actual weight, kg	76.66 ± 12.83	80 ± 12.68	0.224
Body weight loss %	4.14 (2.88; 6.70)	0.00 (0.00; 0.00)	<0.00001
Cachexia, yes (%)	9 (38)	/	
Hemoglobin, g/dl	11.28 ± 2.50	14.07 ± 2.04	0.002
C-reactive protein *, mg/dl	1.19 (0.24; 3.01)	0.30 (0.22; 0.50)	0.134
Albumin, g/dl	3.25 (2.98; 3.53)	4 (3.80; 4)	0.0006
Comorbidities			
Hypertension, n (%)	14 (58)	8 (61)	0.850
Diabetes, n (%)	7 (29)	4 (31)	0.919
Dyslipidemia, n (%)	7 (29)	6 (46)	0.301
Type of cancer			
Pancreas, n (%)	9 (38)	/	/
Colorectal, n (%)	8 (33)	/	/
Gastric, n (%)	7 (29)	/	/
Stage I-II, n (%)	16 (67)	/	/
Stage III-IV, n (%)	8 (33)	/	/

Abbreviations. Body mass index, BMI. * normal range: 0–0.5 mg/dL.

## Data Availability

The data presented in this study are available on request from the corresponding author.
